# A Wearable Device to Measure Bioimpedance and Electrodermal Activity

**DOI:** 10.3390/s26185918

**Published:** 2026-09-19

**Authors:** Jesús Ponce de León, José Ramón Beltrán, David Asiain

**Affiliations:** 1 Escuela Universitaria Politécnica de La Almunia (EUPLA), University of Zaragoza, 50100 Zaragoza, Spain; jponce@unizar.es (J.P.d.L.); dasiain@unizar.es (D.A.); 2 Escuela de Ingeniería y Arquitectura (EINA), University of Zaragoza, 50009 Zaragoza, Spain

**Keywords:** bioimpedance (BiZ), electrodermal activity (EDA), approximate Cole model of skin, AFE AD5941 integrated circuit, wearables, biosignals

## Abstract

Continuous non-invasive health monitoring requires wearable devices capable of capturing synchronized, multi-parametric physiological data without compromising user comfort or signal integrity. However, existing platforms often suffer from insufficient accuracy in complex modalities such bioimpedance. To address these limitations, this paper introduces the Multi-sensor Wearable Wristband Version 2.0 (${\mathrm{MsW}}^{2}$-v2), an optimized, highly integrated platform designed for high-precision measurements. The methodology encompasses the hardware optimization of a previous prototype, integrating a low-leakage switching matrix and dual-bandwidth analog front ends to capture, in a quasi-simultaneous (time-multiplexed) manner, electrodermal activity (EDA) and bioimpedance (BiZ), as well as other physiological signals such as skin temperature, oxygen saturation, heart rate, and inertial motion. System validation was performed through rigorous laboratory calibration against reference impedances and subsequent in vivo testing on a human volunteer in an isolated room with no external stimuli whatsoever. The laboratory results indicated high-precision performance, with the mean absolute errors of the measurements being below 3 Ω in modulus and 0.2° in phase across a wide range of frequencies, from 1 kHz to 200 kHz. Preliminary in vivo benchmarks confirm the functionality of the device. These findings demonstrate that the ${\mathrm{MsW}}^{2}$-v2 successfully bridges the gap between high accuracy and wearable form factors, bringing us to the conclusion that this device could serve as a viable, scalable solution for real-time physiological measurements and biomedical signal monitoring.

## 1. Introduction

This paper presents a new multisensor device capable of quasi-simultaneously measuring electrodermal activity (EDA) and bioimpedance (BiZ). These variables are rarely measured on a single platform, especially in low-power systems. The device also features a position and motion sensor (inertial measurement unit), a pulse oximeter and heart rate monitor, and an infrared temperature sensor. The size, weight, and power consumption [1,2] of the device presented here make it ideal for wearable applications.

### 1.1. Wearable Devices

A wearable device is a ubiquitous, miniaturized, and autonomous computing system designed to be worn continuously on the body, on the skin, or integrated into the user’s clothing. Unlike conventional mobile devices, it operates on the principle of passive and proactive interaction, functioning in the background without interrupting the user’s activity.

#### 1.1.1. Wearable Devices for Health

With the appropriate software embedded, wearable devices are intelligent electronic platforms, usually in the form of accessories (forehead bands, bracelets, patches, or watches), that allow the collection of physiological data from the user and, in general, a more or less detailed analysis of these data, with fewer restrictions than traditional laboratory devices [3]. The development of platforms for recording, monitoring and analyzing physiological signals has continued growing for more than a decade [4] and is now a very active branch of sensor electronics [5]. The range of uses of captured physiological signals has expanded in proportion to the spread of this technology, from the extraction of health-related characteristics to the classification of emotions or moods. More specific applications of physiological signals may include, among others, disease diagnosis [6], remote healthcare [7,8], human–machine interaction (e.g., to increase the autonomy of people with disabilities) [9], rehabilitation processes [10], stress detection [11], monitoring of daily activity [12], personal comfort assessment [13], and emotion detection [14].

#### 1.1.2. Wearable Devices for EDA and BiZ Measurements

Since this paper will focus exclusively on these two variables, it is worth briefly reviewing the current wearable devices capable of taking these measurements. These can be divided into two broad categories: mass-market commercial systems and research-grade devices.

Regarding mass-market systems, the widespread adoption of four-wire bioimpedance measurement at the wrist has been driven by the integration of system-in-package (SiP) sensors, made available through development environments such as Samsung’s SDK (Samsung Electronics Co., Ltd., “Samsung Health Sensor SDK,” Samsung Developer Portal, 2024. [Online]. Available: https://developer.samsung.com/health/sensor/overview.html [Accessed: 3 September 2026]). On the other hand, the Google Pixel Watch (Google LLC, “Google Pixel Watch technical specifications,” Google Pixel Watch Help, [Online]. Available: https://support.google.com/googlepixelwatch/answer/12651869?hl=en [Accessed: 3 September 2026]) and Fitbit (Google LLC, Fitbit user manuals, Google Health Support, [Online]. Available: https://support.google.com/googlehealth/answer/14253977?hl=en [Accessed: 3 September 2026]) are capable of measuring EDA (but not BiZ) continuously.

Unlike general mass-market systems, the most common research-grade devices provide access to the raw data needed for many lines of research. Among these devices, the E4 Empatica wristband (Empatica Inc., “E4 Wristband,” Empatica Research. [Online]. Available: https://www.empatica.com/research/e4/ [Accessed: 3 September 2026]), now discontinued, is probably the best known. On the other hand, for accurate conductance (BiZ) measurement in clinical settings, devices with open specifications—such as the Shimmer3 GSR+ (eBio) (Shimmer Sensing, “Shimmer3 User Guide,” Wireless Sensor Networks Documentation, Rev. 1.15, Shimmer Research, Dublin, Ireland, 2021. [Online]. Available: https://www.shimmersensing.com/support/wireless-sensor-networks-documentation/ [Accessed: 3 September 2026])—are typically preferred.

The device presented in this work would fall into the latter category.

#### 1.1.3. Methodological Comparison

A methodological comparison of the devices mentioned and the MsW^2^-v2 platform can be found in Table 1.

As shown in the table, the Shimmer3 GSR+ (first column) allows external configuration of the input channels to record phasic/tonic EDA along with localized impedance signals by attaching auxiliary galvanic sensors to fingers or limbs. Its successor, the Shimmer3 eBio (second column), is capable of performing tetrapolar BiZ measurements (but not EDA) at a frequency of up to just over 1 kHz. On the other hand, the Empatica E4 and its successor, the Embrace Plus (third column), are considered the analytical standard in the clinical literature. The EDA module measures exosomatic conductance by applying a fixed direct current (DC) through two silver/silver chloride (Ag/AgCl) electrodes. All wearables in this first category allow (direct or indirect) access to raw data.

Among the mass-market devices, the Samsung Galaxy Watch (Series 4 through Ultra, fifth column) uses four contact points (two on the base of the watch and two on the physical buttons that the user must press with the other hand) and injects a multi-frequency AC signal to isolate whole-body impedance. Meanwhile, the Google devices listed (last column) incorporate a continuous electrodermal activity (cEDA) sensor, which uses an ultra-low-power constant-voltage front end to continuously measure phasic responses on the skin of the wrist to map autonomic stress, using proprietary algorithms that filter out the tonic signal. None of these devices allow access to raw data.

As will be detailed below, using time-division multiplexing, the MsW^2^-v2 platform can measure both EDA (low-frequency AC, up to 10 Hz) and BiZ (AC up to 200 kHz) signals. The device can be configured via software for two-wire and four-wire measurements, and the raw data for both EDA and BiZ are available for further analysis and can be cross-referenced with data from other sensors within the same device for more specific purposes.

### 1.2. EDA and BiZ

EDA and BiZ are two different physiological variables used to measure different aspects of the human body. EDA measures changes in the electrical conductance of the skin, changes that are often associated with autonomic nervous system activity and emotional responses such as stress, fear, anxiety or arousal, as well as alterations related to psychological and sleep disorders [15,16]. BiZ, on the other hand, focuses more on aspects associated with body composition. However, both variables can be measured using similar techniques; specifically, both are obtained from the body’s response (impedance) to the flow of a small electric current, which, in turn, opens the possibility of measuring both using the same device.

The wearable device presented in this work allows the measurement of these and other physiological variables. This lightweight, versatile, and energy-efficient device, capable of accurately measuring both EDA and BiZ signals, will be the focus of the present study.

#### 1.2.1. EDA

EDA is directly regulated by the sympathetic nervous system, which is activated by emotions such as stress, fear, excitement or surprise [17,18]. More specifically, EDA measures fluctuations in skin electrical conductance, which is related to sweat gland activity. Activation of the sweat glands causes an increase in sweating, which, in turn, increases the electrical conductance of the skin (or, in other words, decreases its electrical resistance [19]). Thanks to this purely physiological reaction, EDA is widely used in emotion recognition and classification [20,21].

#### 1.2.2. BiZ

BiZ, as the name suggests, measures the impedance of biological tissues. BiZ analysis is a noninvasive technique that measures this electrical property by applying a low-amplitude alternating current and analyzes the electrical response of the body as a function of frequency [22]. BiZ in biological tissues depends on the cellular structure and the composition of extracellular and intracellular fluids. Indeed, human tissues can be modeled as heterogeneous media composed of multiple layers with different electrical properties that determine their behavior in the presence of alternating current [23]. Tissue impedance is a complex quantity, generated from the resistance and capacitance specific to the composition of each layer:

$$Z=R+j\cdot X_{C},$$

where *Z* is the impedance of the biological tissue; *R* is the resistive opposition to the electric current flow due to intracellular and extracellular fluids; and $X_{C}$ is the charge storage capacity of cell membranes, which influences current transmission.

**Figure 1:**
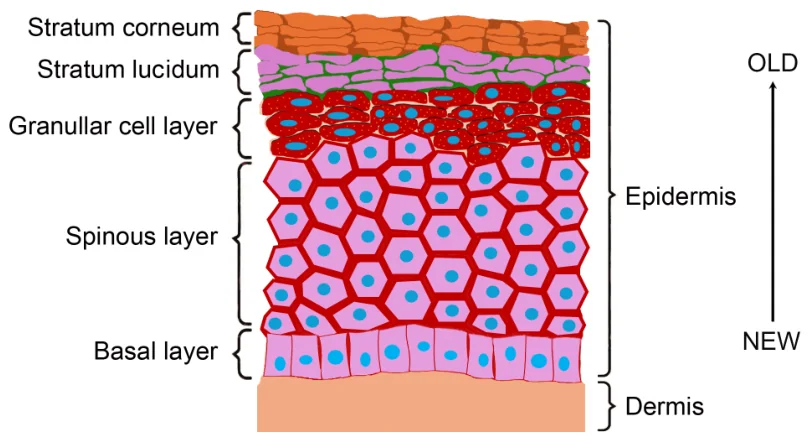
Layers of human skin.

As the electric current penetrates deeper into different tissue layers (see Figure 1), it encounters different resistances and capacitances according to the composition of each layer [23]. As can be seen in the figure, there are five different cell layers in human skin. The outermost (and oldest) one is the stratum corneum, highly resistive due to the presence of dead cells and low amount of water. At increasing depths, we have the stratum lucidum, the granular cell layer, and the spinous layer. The deeper into the skin, the higher the water and electrolyte content, as is the case with intercellular tissue, which causes a decrease in the resistance of skin and an increase in its capacity. The deepest layer of the epidermis is the basal layer, where new cells are generated to replace the upper layers as they develop. Just below the basal layer is the dermis, and beneath that is muscle tissue (not shown in the figure). BiZ is generally measured at different frequencies to obtain information on the distribution of body fluids and tissue structure. At low frequencies the current flows mainly through the extracellular space, whereas at higher frequencies it can pass through cell membranes, allowing a more complete evaluation of the biological environment [24].

On the other hand, BiZ also allows the detection of changes in arterial volume or diameter, since when an artery expands or contracts, the amount of blood flowing through it changes, which, in turn, causes a change in the impedance to be measured. In general, the greater the blood volume, the lower the impedance [25]. These changes in arterial volume or diameter may also reflect the activity of the sympathetic nervous system and, consequently, give information about the emotional state of the individual, reinforcing and enriching the data obtained by EDA. Acute sympathetic stimuli such as exercise or electrical stimulation itself, mental stress or exposure to cold can cause a significant reduction in arterial diameter (mainly centered in the extremities). Although these changes may be small or vary depending on the artery and the stimulus, BiZ presents an interesting potential to detect them [26,27].

Numerous equivalent circuit models have been proposed for the complex impedance of skin tissue [28,29,30]. In our case, we selected the simplified Cole model [31], as it reproduces the tissue behavior with closely spaced electrodes (in our case, the electrodes are located on the fingertips) and over a wide frequency range (up to 10 kHz) [32]. This model is mathematically simple and does not require significant computing power for implementation in wearable applications.

#### 1.2.3. The Approximate Cole Model

The Cole model is one of the most widely used formulations to describe the electrical behavior of biological tissues over a wide range of frequencies. It is based on the observation that the impedance of biological tissues does not follow a strictly ideal capacitive–resistive behavior but exhibits a characteristic dielectric dispersion [24]. The Cole model is described by the following expression:

(1) $$Z\left(f\right)=R_{\infty }+\frac{R_{0}-R_{\infty }}{1+{\left(j2\pi f\tau \right)}^{1-\alpha }}$$

In this equation, $R_{0}$ is the resistance at low frequency, representing the flow of current through the extracellular fluids; $R_{\infty }$ is the resistance at high frequency, when current passes through both the extracellular and intracellular compartments; τ is the characteristic time constant of dielectric dispersion; and α is the dielectric dispersion parameter (0<α≤1), representing the non-uniform distribution of relaxation times in biological tissues.

**Figure 2:**
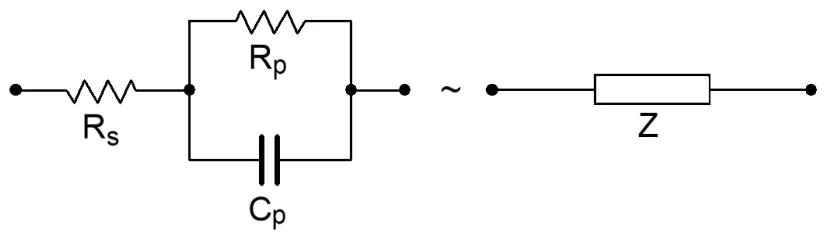
Simplified Cole model of tissue impedance, $R_{s}+R_{p}\left|\right|C_{p}\to Z$.

However, in its simplified form, this model can be reduced to a small series resistor ($R_{s}$) followed by a larger resistor ($R_{p}$) and a capacitor ($C_{p}$) in parallel [24], as shown in Figure 2.

In this case, the complex equivalent impedance can be written as

(2) $$Z\left(f\right)=R_{s}+\frac{R_{p}}{1+j2\pi fR_{p}C_{p}}$$

From this expression, it is easy to obtain both the real part (*ℜ*) and the imaginary part (*ℑ*) of the impedance, these being, respectively,

(3) $$ℜ\left(f\right)=R_{S}+\frac{R_{p}}{1+{\left(2\pi fR_{p}C_{p}\right)}^{2}}$$

and

(4) $$ℑ\left(f\right)=-\frac{R_{p}^{2}2\pi fC_{p}}{1+{\left(2\pi fR_{p}C_{p}\right)}^{2}}$$

Finally, the modulus (|Z(f)|) and phase ($\Phi_{Z(f)}$) of the complex impedance are easily obtained from these expressions, that is,

(5) $$\left|Z\left(f\right)\right|=\sqrt{ℜ{\left(f\right)}^{2}+ℑ{\left(f\right)}^{2}}=\frac{\sqrt{{\left\{R_{s}\left[1+{\left(2\pi fR_{p}C_{p}\right)}^{2}\right]+R_{p}\right\}}^{2}+{\left\{R_{p}^{2}2\pi fC_{p}\right\}}^{2}}}{1+{\left(2\pi fR_{p}C_{p}\right)}^{2}}$$

and

(6) $$\Phi_{Z(f)}=arg\left\{\frac{ℑ(f)}{ℜ(f)}\right\}=arg\left\{\frac{-R_{p}^{2}2\pi fC_{p}}{R_{s}\left[1+{\left(2\pi fR_{p}C_{p}\right)}^{2}\right]}\right\}$$

As will be seen later, this simplified mathematical model has been used to generate a set of laboratory impedances (covering the usual full range of impedances of human tissue) and thus to fine-tune our measurement device.

### 1.3. Organization of the Paper

This work details the development of MsW^2^-v2, a wearable platform for the capture of biomedical signals. In Section 2, the developed device is detailed in terms of hardware (Section 2.1, Section 2.2, Section 2.3 and Section 2.4) and software (Section 2.5), and the EDA and BiZ measurements are explained in Section 2.6. In Section 3, we explain the methodology of the process of calibration (Section 3.1 and Section 3.2) and the main results (Section 3.3) of the device measurement, discussed in Section 4. Finally, Section 5 presents the conclusions and future lines of research.

## 2. Device Description

**Figure 3:**
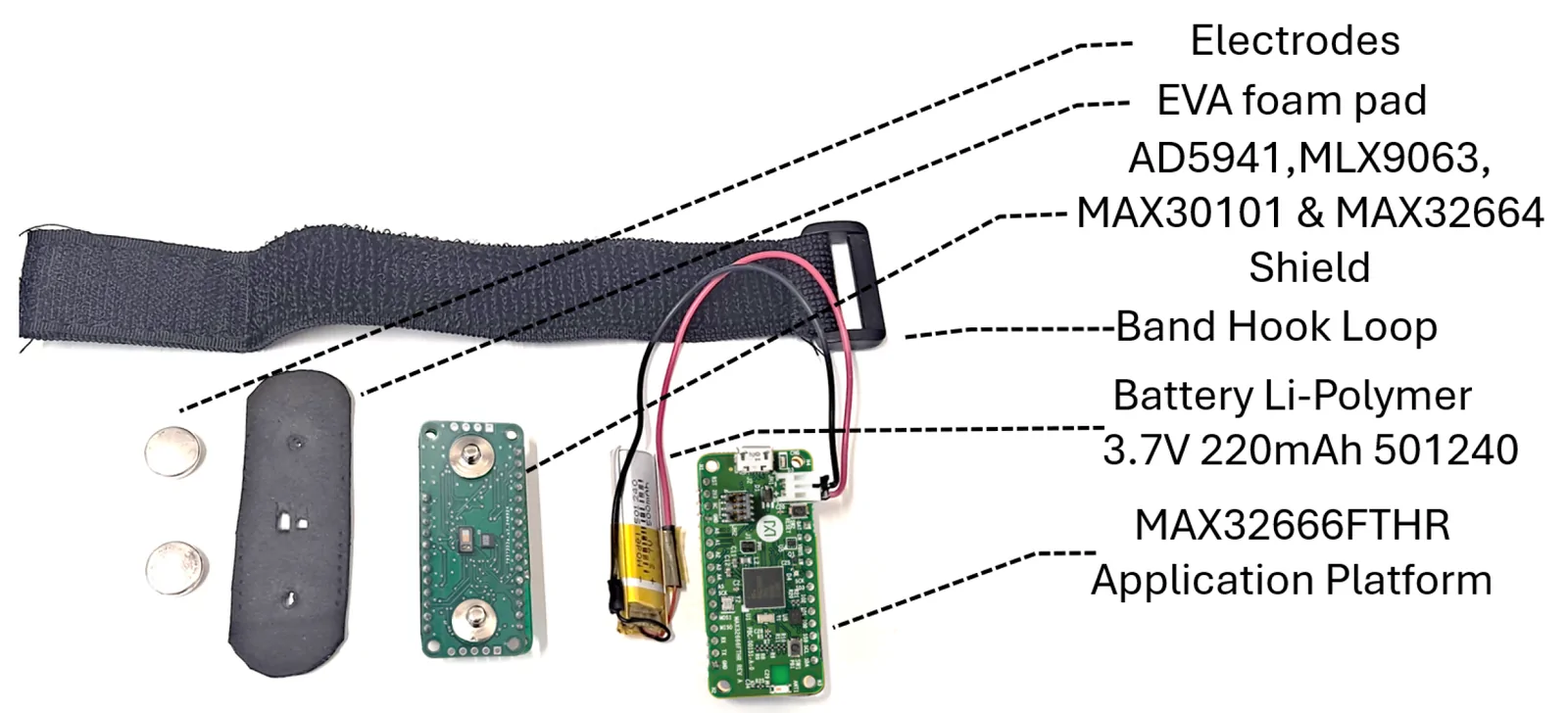
Real image of the MsW^2^-v2 prototype. Affective Labs, La Almunia, Zaragoza, Spain.

The device presented in this work is a natural evolution of our previous Multi-sensor Wearable Wristband (MsW^2^) [1,2], which has undergone a technological upgrade and now includes BiZ measurement functionality, a capability that the previous model lacked. Although this paper will focus on EDA and BiZ measurements, MsW^2^-v2 is capable of measuring other physiological signals such as body temperature, oxygen saturation, and HR, as well as the user’s position and movements as captured by a complete IMU system. The device hardware is distributed across a double PCB, with a total area of 0.9 × 2.0 inches (2.3 × 5.1 cm). It includes an EDA and BiZ sensor, an oxygen saturation sensor, a temperature measurement sensor, and an inertial system. The details of the PCB and the different devices it includes can be seen in Figure 3.

### 2.1. Hardware Description

**Figure 4:**
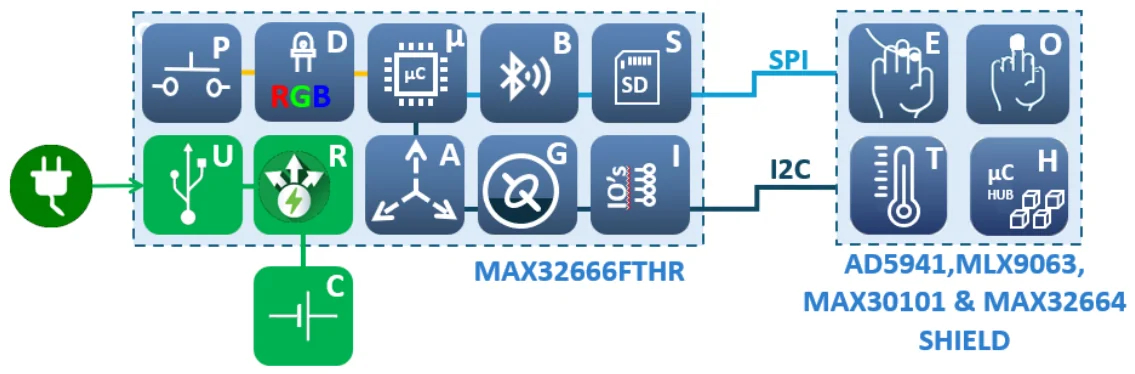
Conceptual block diagram of the MsW^2^-v2 device.

The developed prototype uses a MAX32666 (https://www.analog.com/media/en/technical-documentation/data-sheets/max32665-max32666.pdf. Accessed on 14 September 2026) FTHR kit. It incorporates a small 3.7 V lithium battery whose charging/discharging is controlled by the MAX1555 (https://www.analog.com/media/en/technical-documentation/data-sheets/max1551-max1555.pdf. Accessed on 14 September 2026) battery charge management circuit, optimized for low-power wearable devices. The BMI160 (https://community.bosch-sensortec.com/knowledge-base-pg631enp/post/bmi160-series-imu-design-guide-31X8YTuWV9btkRM. Accessed on 17 September 2026) is a low-power inertial device capable of measuring acceleration and angular velocity. The use of gyroscopes/accelerometers is important in wearable devices because, in addition to sending information about the user’s position and movements, it allows one to correct (through a simple correlation) the artifacts caused by physical activity in other monitored variables, such as, in our case, EDA and BiZ signals. The MAX32666 FTHR kit also incorporates a Bluetooth Low Energy module (BLE version 5). In addition to all the detailed systems, a MAX32666 dual-core microprocessor is responsible for managing the complete set of measurements, which can be stored on a microSD card or sent for external processing via Bluetooth. The block diagram can be seen in Figure 4.

As can be seen in Figure 4, the MAX32666 board is completed with a self-made shield, consisting of an AD5941 (https://www.analog.com/media/en/technical-documentation/data-sheets/ad5940-5941.pdf. Accessed on 14 September 2026) (EDA and BiZ sensor), an MLX90632 (also known as MLX9063) (https://www.melexis.com/en/documents/documentation/datasheets/datasheet-mlx90632. Accessed on 14 September 2026) (FIR temperature sensor) and a MAX30101 (https://www.analog.com/media/en/technical-documentation/data-sheets/max30101.pdf. Accessed on 14 September 2026) (pulse oximeter and heart rate sensor) accompanied by its own microprocessor, a MAX32664 (https://www.analog.com/media/en/technical-documentation/data-sheets/MAX32664.pdf. Accessed on 14 September 2026). These circuits communicate with the main board via I^2^C and SPI connections.

All the integrated circuits included in the ${\mathrm{MsW}}^{2}$-v2 prototype, their main functions, and their most notable technical characteristics are summarized in Table 2. The following subsections will explain the different systems that measure each of the physiological signals that our device is capable of monitoring, beginning with the measurement of EDA and BiZ, which is the primary focus of this work.

### 2.2. Selection of EDA and BiZ Measurement Hardware

To evaluate the capabilities of the developed platform, EDA and BiZ were established as the primary target biomedical signals. The hardware selection process followed a strict benchmarking methodology based on three design criteria essential for wearable applications: low power consumption, high integration, and minimal external hardware requirements.

Four commercial analog front ends (AFEs) meeting these criteria were analyzed: the AD5941 (Analog Devices, AD, Wilmington, MA, USA), the MAX30009 (Maxim/Analog Devices, M/AD, Wilmington, MA, USA), and the AFE4500 and AFE4300 (Texas Instruments, TI, Dallas, TX, USA). Other market alternatives, such as the AMS OSRAM AS7038RB (ams-OSRAM AG, Premstaetten, Styria, Austria), were excluded from this methodological evaluation because their primary architecture is optimized for SpO_2_ and heart rate (HR), requiring extensive external circuitry to achieve comparable EDA and BiZ accuracy. Table 3 synthesizes the comparative technical specifications that guided the selection process.

Based on this comparative analysis, the AD5941 AFE was selected as the core measurement unit for this work. This choice is methodologically justified by the following technical advantages:

- Measurement Versatility: It allows the integration of EDA, BiZ, ECG, and electrochemical sensing within a single-chip architecture.
- Electrode Configuration: It supports both two-wire setups and four-wire configurations. The latter is critical to isolate the excitation and measurement loops, thereby minimizing measurement errors induced by contact resistance and electrode polarization.
- Signal Processing and Autonomy: It features an expanded frequency range, versatile excitation modes, and hardware-level complex variable measurement capabilities.
- Efficiency: The inclusion of an embedded autonomous sequencer significantly reduces microcontroller overhead and power consumption, fulfilling the constraints required for robust research and wearable development.

### 2.3. AFE ADF5941

**Figure 5:**
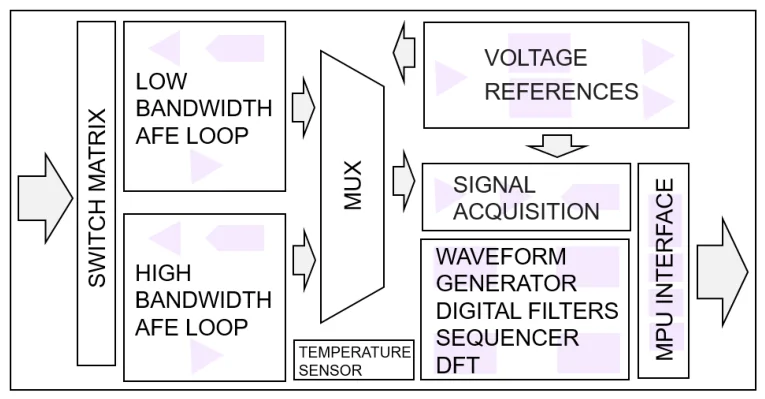
AD5941 block diagram. The signals go from left (input) to right (output). The acquired signal can be analyzed by either the low-frequency loop (EDA) or the high-frequency loop (BiZ). An analog multiplexer determines which loop to use. The acquired signal is filtered and sent to the output.

The AD5941 features an internal architecture composed of multiple functional blocks, both analog and digital, that work together to enable high-precision impedance and electrochemical signal measurements. These blocks include amplifiers, A/D and D/A converters, digital filters, and a signal generator, as well as a switching matrix and an internal sequencer, among others. These functional blocks can be seen in Figure 5.

In summary, the AD5940/AD5941 chip is a highly flexible, low-power analog front end (AFE) designed for advanced electrochemical and BiZ measurements. It features a versatile switching matrix that supports multiple electrode configurations, alongside dual low-bandwidth and high-bandwidth AFE loops powered by integrated DACs to precise-handle currents across a wide frequency range. High-accuracy data acquisition is driven by a multi-channel 16-bit ADC with an anti-aliasing filter, while a dedicated DFT module directly calculates complex impedance in hardware to offload processing from the microcontroller. Finally, the chip optimizes power efficiency and system integration through an automated programmable sequencer with SRAM, an SPI-based communications module, and an internal temperature sensor for thermal drift calibration.

### 2.4. Other Biosignal Sensors

In the next subsections, we provide a brief explanation of the other biomedical sensors integrated into the device: the inertial measurement unit (included in the MAX32666 board), the pulse oximeter (and heart rate sensor), and the skin temperature sensor (both integrated into the developed shield).

#### 2.4.1. Inertial Measurement Unit

The BMI160 is a six-axis (three-axis accelerometer and three-axis gyroscope) inertial measurement unit (IMU) that combines a 16-bit accelerometer and gyroscope. Key specifications include low power consumption, programmable acceleration ranges (from ±2 g to ±16 g) and gyroscope ranges (from ±125°/s to ±2000°/s), and communication interfaces such as I^2^C and SPI. Its low-power design makes it suitable for mobile applications and, in particular, for wearable devices.

#### 2.4.2. Blood Oxygen Saturation (SpO_2_) and Heart Rate (HR) Monitoring

As noted before, the pulse oximeter and heart rate monitoring sensor is a MAX30101, a high-sensitivity pulse oximeter and heart rate sensor for wearable health monitoring. It also supports estimated blood pressure monitoring. The MAX30101 is an integrated module that includes internal LEDs (red and IR for sensing and green for ambient-light cancellation), photodetectors, and other optical elements, with low-noise electronics. The monitored data are sent to a MAX32664 micro-controller. The MAX32664 seamlessly enables any desired sensor functionality and includes communication with Maxim’s optical sensors. It can deliver raw or processed data to the outside world through a fast-mode slave I^2^C interface.

#### 2.4.3. Temperature Sensor

Finally, the developed shield incorporates the MLX90632, a small non-contact infrared temperature with a 50º field of view (FoV). The manufacturer has previously calibrated this device to achieve high accuracy by storing its configuration in an EEPROM memory. Its specific encapsulation reduces thermal disturbances. The sensor has the necessary electronics to digitize and filter the temperature information, which is stored in a RAM memory to be sent to the microcontroller. In addition, the MLX90632 can measure its own temperature. The temperature sensor uses the I^2^C protocol, which, in our prototype, operates at 1.8 V. This device has different presentations, standard and medical, whose most significant difference is in their accuracy. The medical version is factory calibrated with an accuracy of ±0.2 °C and has a resolution of 0.01 °C. In addition, it has three operating modes: idle mode, step mode and continuous mode. The difference between the latter two is the measurement frequency: the step mode performs a measurement only when requested by the microcontroller.

### 2.5. Software Description

The firmware was developed on the Analog Devices MSDK platform (https://analogdevicesinc.github.io/msdk/USERGUIDE/. Accessed 26 September 2025), which provides the necessary software, including peripheral drivers, libraries, and example code. The firmware does not use an operating system and utilizes a bare-metal Bluetooth example as its base. The drivers for the AD594x (EDA) and MLX9063 (infrared temperature sensor) were ported to the platform. For the MAX32664 hub processor, which communicates with the MAX30101 (SpO_2_ sensor), a completely new implementation (not a port) was used.

All data transfers are performed via interrupts except for the MLX9063, which uses synchronous polling, synchronized with the AD594x interrupt transfer. The firmware generates three data streams that can be transmitted via Bluetooth or stored on the SD card. The first stream contains EDA, BiZ and temperature data at 4 Hz; the second stream contains SpO_2_ data at 100 Hz; and the third stream contains inertial sensor data at 50 Hz.

### 2.6. EDA and BiZ Measurements

The AD5941 can quickly switch between measurement modes (e.g., switching between measuring BiZ and other parameters) thanks to its sequencer. This device can be easily integrated with other analog front ends. For example, it can be coordinated with specific devices for ECG measurement (such as the AD8233) and use the same electrodes to perform alternate measurements of BiZ, EDA, and ECG, due to the rapid reconfiguration capability provided by its integrated sequencer and communication via IRQs with the microcontroller [33,34]. In this work, we will focus on the AD5941’s capacity for EDA and BiZ measurements. The AD5941’s operating frequency is 4 Hz, which means that, in the simplest configuration (the one used here), we have 250 mS to switch between EDA and BiZ measurements. As will be seen, this limits the bandwidth for the in vivo measurements presented in Section 3.3.2.

#### 2.6.1. EDA Measurement

The AD5941 is optimized for EDA measurement, using a low-power loop and complying with IEC 60601 medical safety restrictions [35], which limit direct currents (<10 μA DC below 1 kHz). To do this, over a limiting resistance, it applies a low-frequency (100 Hz) and low-amplitude (a few hundred mV) sinusoidal excitation using its DAC and potentiostat amplifier [1,34].

In the usual two-electrode configuration, one electrode applies the electrical stimulus and the other measures the resulting current through the transimpedance amplifier (TIA). Due to the wide impedance range of the skin (from tens of kiloohms to a few megaohms), the AD5941 is able to adjust the resistance of the TIA amplifier to optimize sensitivity and accuracy according to skin conditions [36]. Its 16-bit ADC and integrated digital filters enable accurate measurements even in high-noise conditions.

In addition, the AD5941 is capable of measuring both the resistive and capacitive parts of skin impedance using alternating current, which can provide additional information about epidermal properties and changes due to sweating. This information is calculated directly using the chip’s internal discrete Fourier transform (DFT).

#### 2.6.2. BiZ Measurement

As has been explained, for BiZ measurements the AD5941 uses the high-bandwidth loop, as excitation frequencies can easily reach hundreds of kilohertz. In particular, the AD5941 can generate a sinusoidal signal up to 200 kHz and accurately measure the magnitude and phase of the resulting impedance thanks to its high-speed TIA and DFT module. To measure BiZ, a known alternating signal is typically applied between electrodes placed on the human body, and the electrical response of the tissue is then measured.

As previously moted, to ensure compliance with IEC 60601-1 safety standards [35], the AD5941 internally limits the maximum excitation voltage (∼1.2 V peak-to-peak voltage), and external resistors are used to prevent excessive currents in the event of a short circuit or low impedance [1]. Furthermore, series isolation capacitors must be used to block any DC components, thereby complying with regulatory requirements for safe leakage current [33,34].

## 3. Methodology: Calibration and Experiments

Our last aim is to use the MsW^2^-v2 for the acquisition and analysis of biometric signals. The measurement of the other variables has already been validated [1,2], so in this study we will focus on optimizing the device for the simultaneous (or quasi-simultaneous) measurement of EDA and BiZ. These measurements will first be performed on a set of laboratory impedances (in vitro measurements) and then on a volunteer subject (in vivo measurements; see Institutional Review Board at the end of this work).

The EDA and BiZ measurement process is subject to several errors due to both the device itself and the electrodes. Of these errors, it is feasible to accurately estimate those due to the internal parasitic impedances of the measuring device (inherent to any measurement process). Regarding parasitic effects from the wiring (both from the measurement cable itself and from electrode contact with the skin), these appear in the in vivo measurements and are much more difficult to estimate. These effects could be minimized by performing a four-wire measurement, perfectly feasible with the AD5941, but in this case this method was discarded as it was considered excessively cumbersome. As will be seen later, the in vivo measurements were performed at frequencies up to 1 kHz, which places us at the threshold of EDA and little else (the first layers of the skin, dominated by the stratum corneum). Under such conditions, the influence of these parasitic elements is not significant, since the exact impedance value is not usually as important as its variation.

However, the developed device is capable of performing measurements across a wide frequency range (up to 200 kHz). To achieve high-precision results in the laboratory (that is, not over biological tissues), a set of six impedances was constructed, covering the typical impedance range of the skin’s surface layers under varying humidity conditions. The parasitic inductor and capacitor present on the AD5941 PCB itself and the measurement loop were then estimated through a calibration process, which will be detailed below.

### 3.1. BiZ Measurement Loop Parasitic

**Figure 6:**
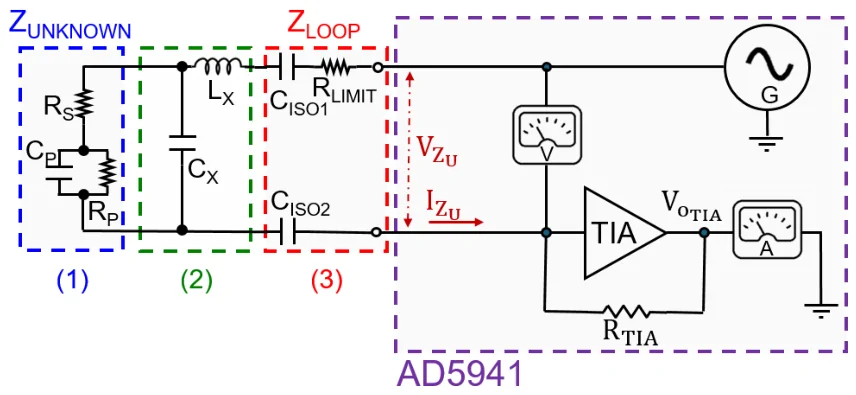
BiZ measurement and calibration circuit. From left to right: (1) Cole model $Z_{UNKNOWN}$, in blue. (2) Parasitic impedances of AD5941 PCB. (3) External $Z_{LOOP}$ impedance. Finally, the AD5941 measurement model, in purple. See text for details.

The measurement and calibration circuit is summarized in Figure 6.

In this figure, from right to left, the following can be seen. Framed in purple is the AD5941, consisting of a voltage excitation source and a transimpedance amplifier (TIA), whose gain is controlled by the $R_{TIA}$ resistor. This device measures a voltage proportional to the current flowing through the measurement loop, in the figure divided in three different sections, numbered (1) through (3).

This loop consists of (3), marked in red in the figure: the external impedance $Z_{LOOP}$, composed of a current-limiting resistor $R_{LIMIT}$ (for compliance with IEC 60601-1 safety standards) and the isolation capacitors $C_{ISO1}$ and $C_{ISO2}$. The electrodes are placed at the ends of the impedance to be measured (either laboratory impedances or human body impedances). However, the PCB of the measurement device itself has parasitic impedances, which appear in green in the figure and are marked as (2): an inductance $L_{X}$ in series with $C_{ISO1}$ and $R_{LIMIT}$ and a capacitor $C_{X}$ in parallel with $Z_{UNKNOWN}$. These parasitic reactances are negligible at DC or very low frequencies (EDA), but their global significance increases significantly as the frequency of the excitation signal rises. Finally, in blue, comes (1) $Z_{UNKNOWN}$, represented in the figure by the laboratory impedances (based on the approximate Cole model of human tissues). In the case of in vivo measurements, $Z_{UNKNOWN}$ is the impedance of the skin (BiZ or EDA) of the test subject. In this case, the Cole model is still applicable because it is a very superficial measurement, on the first layers of the *stratum corneum*, and as stated in Section 1.2.2, each layer of the skin is characterized (basically) by a single resistance in parallel with a single capacitor.

In this way, it is easy to see that

(7) $$Z_{S}=Z_{R_{LIMIT}}+Z_{ISO_{1}}+Z_{ISO_{2}}+Z_{L_{X}}=Z_{LOOP}+Z_{L_{X}}$$

In this equation, $Z_{S}$ is the impedance of the series connection of $R_{LIMIT}$, $C_{ISO_{1}}$, $C_{ISO_{2}}$ (named $Z_{LOOP}$) and the inductance $L_{X}$.

If $Z_{C_{X}}$ is the impedance of the parasitic capacitor and $Z_{UNKNOWN}$ is the target impedance, the total impedance measured by the system $Z_{T}$ can be written as

(8) $$Z_{T}=Z_{LOOP}+Z_{L_{X}}+\frac{Z_{C_{X}}\;Z_{UNKNOWN}}{Z_{C_{X}}+Z_{UNKNOWN}}$$

From Equation (8), it can easily be deduced that, under short-circuit conditions (where $Z_{UNKNOWN}=Z_{C_{X}}=0$), the measured impedance $Z_{T}$ coincides with the series of $Z_{LOOP}$ and $Z_{L_{X}}$. Since the impedance $Z_{LOOP}$ is known, this allows us to characterize $Z_{L_{X}}$. The capacitor $C_{X}$ is inferred by an indirect measurement, through measurements of very large impedances of known value (see next section). The value of the parasitic capacitance is then fine-tuned to minimize errors in these measurements.

Again from Equation (8), we can see that the impedance to be characterized, $Z_{UNKNOWN}$, is

(9) $$Z_{UNKNOWN}=\frac{Z_{C_{X}}\cdot \left(Z_{T}-Z_{LOOP}-Z_{L_{X}}\right)}{Z_{T}-Z_{LOOP}-Z_{L_{X}}-Z_{C_{X}}}$$

Therefore, once these parasitic components $L_{X}$ (25 μH) and $C_{X}$ (0.875 pF) are appropriately estimated and fine-tuned, the system will be ready for general impedance measurement. Device validation will be carried out in two steps. In the first step, the accuracy of the impedance measurement will be evaluated over a wide frequency range (up to 200 kHz). Once this accuracy is verified, the aim is to evaluate the dual measurement capability of the MsW^2^-v2 (EDA and BiZ). The first test will be performed with a set of laboratory impedances designed to cover the basic characteristics of skin (in vitro measurements). Finally, the aim will be to test the device on human skin, using EDA and BiZ measurements at low frequencies (up to 1 kHz) on a volunteer subject (in vivo measurements).

### 3.2. Laboratory Impedances and Fine-Tuning Process

In order to verify the accuracy of in vitro measurements, a set of impedances with known values is required. As noted before, in our case, a set of six impedances ($Z_{1}$ to $Z_{6}$) has been prepared, modeled according to the simplified Cole model (Section 1.2.3), using an Rs–(Rp||Cp) topology [24]. This configuration is sufficiently accurate to adequately characterize the skin’s surface tissue (stratum corneum) for the in vivo measurements. On the other hand, these impedances can be used to perform a fine-tuning the parasitic of the PCB.

The literature [28,37,38,39,40] confirms that skin impedance values can vary considerably between individuals depending on age, sex, fitness level, lifestyle, and other factors. In addiction, depending on the area of the body where the electrodes are placed and the individual’s perspiration level, the measured impedances can also vary significantly. As shown in various studies, the resistive component of the first layers of skin can range from several MΩ to a few hundred kΩ [38,39]. Meanwhile, the capacitive component of the skin varies from hundreds of nF to values close to 1 nF or lower [28,40]. The series resistance of the model is, in all cases, much lower than the aforementioned values. In general, the resistive component of the skin increases as skin moisture decreases, while the capacitance behaves inversely. Obviously, the total impedance of the model decreases with frequency [28,38,39,40].

Taking all these factors into account, the values of the three components of the Cole model have been chosen to cover different working conditions, as can be seen in Table 4. In summary, $R_{s}$ varies from 1 kΩ to 150 Ω, $R_{p}$ can reach 3 MΩ starting from 220 kΩ, and $C_{p}$ ranges from 1 nF to 100 nF. As can be observed, the values of these components correspond to the orders of magnitude mentioned above.

The components were soldered onto an epoxy fiberglass breadboard PCB using a fine-tip soldering iron. This choice was made deliberately as an alternative to using a solderless breadboard, with the aim of minimizing possible errors arising from false contacts, parasitic inductances, and resistance variations due to unstable connections. The use of a soldered board ensures greater electrical stability and repeatability in measurements (in vitro test).

Once the calibration and adjustment process of the device had been completed and its error had been evaluated by repeatedly measuring the set of impedances $Z_{1}$ to $Z_{6}$ at different frequencies (from 1 kHz to 200 kHz), EDA and BiZ measurements were taken on a subject in order to validate the results and measurements under laboratory conditions (in vivo measurements).

The results of both experiments are detailed next.

### 3.3. Experiments

As mentioned before, the MsW^2^-v2 device has been tested in two different situations. In the first, in order to optimize its measurement accuracy, it has been tested, calibrated and adjusted by measuring a series of laboratory impedances (in vitro measurements, Section 3.3.1). Following this process, the system has been tested on living tissue (in vivo measurements, Section 3.3.2).

#### 3.3.1. In Vitro Measurements

To obtain the error in the measurement of our device, it is first necessary to know as accurately as possible the actual values of the laboratory impedances ($Z_{1}$ to $Z_{6}$) that have been manufactured for the calibration process, which is not immediate given the tolerance of the different components used. To this end, a measurement procedure has been designed that is affordable for almost any laboratory. First (see again Figure 2), the value of the resistance $R_{S}$ is measured using a precision ohmmeter (Fluke). Next, a Gwinstek MSO-2204EA digital oscilloscope with an integrated signal generator is used. Since these instruments used do not provide certified-grade uncertainty, the best way to demonstrate the accuracy of the results is to consider the measurements from them to be correct and to calculate the Type A uncertainty using the experimental measurements taken with the MsW^2^-v2, as will be shown later.

The signal generator of the oscilloscope is used to excite a set of impedances $Z_{1}$ to $Z_{6}$ with a voltage value $V_{s}$. Taking these six impedances as voltage dividers ($R_{S}$ and $R_{P}$||$C_{P}$), it is possible to measure the current flowing in each case ($I_{Z_{U}}$) by measuring the voltage value across $R_{S}$ and using the previously measured $R_{S}$ value (Ohm’s law). With this current value, the total impedance value $Z_{T}$ (see again Equation (8) and Figure 6), is now easily obtained:

$$Z_{T}=\frac{V_{Z_{U}}}{I_{Z_{U}}}=\frac{V_{ex}}{I_{Z_{U}}}=\frac{V_{ex}}{V_{0_{TIA}}/R_{TIA}}=\frac{V_{ex}}{V_{0_{TIA}}}R_{TIA},$$

where $V_{ex}$ is the excitation voltage value of AD5941.

The measurements of this set of laboratory impedances were repeated a total of 10 times for each of a total set of 40 different frequencies ranging from 1 kHz to 200 kHz. By taking measurements at these same frequencies with the developed platform, both results can be statistically compared using the mean value $\bar{Z}$ for each frequency, the standard deviation *s* as a measure of dispersion with respect to the mean, and finally the uncertainty *u*. Concretely,

(10) $$\bar{Z}=\frac{1}{n}\sum_{i=1}^{n}Z_{i}$$

(11) $$s=\sqrt{\frac{1}{n-1}\sum_{i=1}^{n}{\left(Z_{i}-\bar{Z}\right)}^{2}}$$

(12) $$u=\frac{s}{\sqrt{n}}$$

The entire dataset has been summarized and is presented in Table 5. In that table, we can see, for each of the laboratory impedances $Z_{1}$ to $Z_{6}$, an average of the values obtained across four frequency bands that cover the entire spectrum of excitation frequencies in 50 kHz intervals. For each impedance and in each frequency range, we can see the average magnitude and phase obtained, the absolute error for magnitude and phase (comparing the data measured with our device to the impedance measurements obtained using the oscilloscope), and finally the Type A uncertainty, again for magnitude and phase.

To supplement the data presented in Table 5, the maximum absolute error across the 2400 measurements is slightly less than 64 Ω (the relative error for this measurement is 0.87%). As for phase, the maximum deviation of the set of measurements from the expected value is 0.2°.

#### 3.3.2. In Vivo Preliminary Measurements

As can be seen in Figure 3, the developed device features electrodes located on the wrist. In addition to this default configuration, the MsW^2^-v2 includes a connector for electrode extension (two or four electrodes), which can be placed anywhere on the body using a long enough wire. To avoid artifacts caused by poor electrode connections, the most common use of the platform involves placing the electrode extension on the fingertips of the non-dominant hand. We have chosen this same configuration for the in vivo measurements, as it is very simple and produces minimal artifacts.

On the other hand, EDA and BiZ measurements are performed by stimulating the skin with a signal of fixed amplitude and variable frequency. The measurements are then time-multiplexed.

Although the device was designed to perform BiZ measurements, it has not been possible to calibrate it specifically for use on biological tissue due to the lack of reference equipment with which to compare the results obtained in vivo, that is, in a test subject. Using the results obtained during the device calibration described in the previous section, the only remaining parasitic influence should correspond to that of the connection between the electrodes and the skin. To minimize the effects of this parasitic influence (ruling out the four-wire measurement, excessively cumbersome), the excitation signal frequency must be kept as low as possible. Combining this fact with the time-division multiplexing mentioned in the previous paragraph, and given that the sole objective of the test is to verify that the device is capable of performing BiZ measurements, we chose to measure a total of five frequencies at regular intervals (225 Hz) between 100 Hz and 1 kHz. At the lower frequencies in this range, a response similar to EDA is expected. At higher frequencies, the response should vary, but throughout this range, human skin is primarily dominated by the stratum corneum [32], so deeper tissues are excluded from this part of the experiment.

The in vivo measurement was conducted on a volunteer subject who had been properly informed of the scope of the experiment (see Institutional Review Board at the end of this paper) over a 15-min period. The subject is sitting in an ergonomic chair, with both arms resting on the same table where the measuring device is located. The electrodes (the same as those used in the Empatica E4 wristband: silver-plated brass dry-contact metal electrodes) were placed between the index and middle fingers of the non-dominant hand, and during the test, the subject remained as still as possible (to minimize muscle movements and reduce mechanical noise in the signals), sitting in a dimly lit room with no other visual or auditory stimuli. In this situation, the body may react in various ways, including relaxation, boredom, or stress, or even alternate between several of these sensations. In any case, it is expected that over a long enough period, changes in the sympathetic nervous system will elicit a response in BiZ that can be measured by the device.

**Figure 7:**
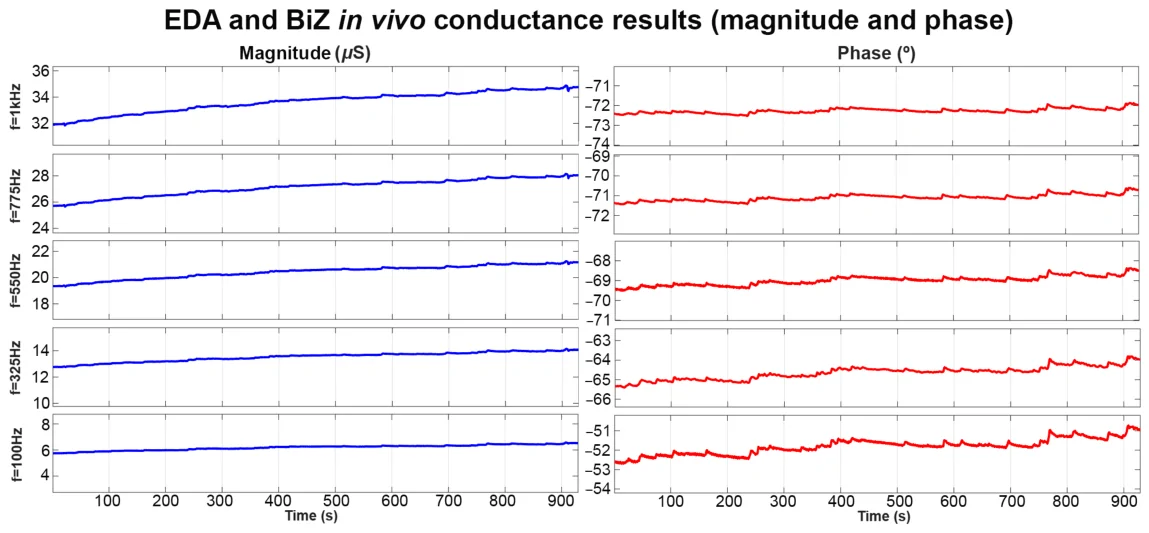
Time evolution of conductance values (magnitude and phase) for 5 different measurement frequencies. In vivo measurements.

The recorded signals are shown in Figure 7. In order to facilitate physiological interpretation, the captured signal values are shown in magnitude (left) and phase (right). Magnitude is given as conductance (in μS) instead of resistance, as is commonly used in EDA signal measurements, while phase is depicted in degrees (°).

These results, along with those related to the in vitro measurements and the overall performance of the device, will be discussed in the following section.

## 4. Discussion

This section will discuss the highlights of the results obtained from the experimental measurements of the developed platform, as well as general issues regarding its performance.

### 4.1. In Vitro Results

With regard to the in vitro results, both the accuracy and repeatability of the measurements taken with the developed wristband are very high. The averaged uncertainties in the measurement of the problem impedance magnitude are less than 4 ω across the four frequency bands under study. With regard to phase, the uncertainties are less than 0.02 degrees in the worst-case scenario. Therefore, we can conclude that the accuracy of our system is very high in the range of 1 to 200 kHz. However, the calibration process is time-consuming and device-dependent. However, although promising, these results should be considered preliminary, since the same set of impedances was used both to tune the circuit and to validate its results.

### 4.2. In Vivo Results

The in vivo results show general behavior consistent with the literature. It can be observed that at lower frequencies the recorded conductance is also lower. This is because at low frequencies, alternating current is more affected by the stratum corneum and the capacitance of the skin, which translates into higher impedance and therefore lower conductance. As the frequency increases, the current can penetrate more easily into deeper layers of tissue. As a result, the total impedance is reduced and so the measured conductance increases.

The temporal evolution of the in vivo results shows an increase in conductance. This is due to increased activity of the sweat glands, confirmed on the part of the volunteer subject.

### 4.3. Overall Performance

Technology, in terms of both sensors and MPUs, is advancing rapidly. The device here presented uses state-of-the-art sensors for EDA and BiZ measurement, SpO_2_, temperature, as well as a complete inertial system (IMU). All these sensors are managed by a high-performance dual-core processor. A low-power design has been implemented that allows all these measurement systems to coexist on a very small (1.8 square inches) and lightweight (25 g battery included) board, making it ideal for wearable applications.

The lack of this type of multi-signal platforms for wearable applications is a serious handicap in research. Their development could lead to significant advances in applications, for example, those related to healthcare. For instance, focusing only on the capacity of our platform in two- and four-wire measurement, the potential applications of BiZ measurement are multiple: fluid retention detection, fluid balance assessment, sports activity monitoring, general activity recognition, physiological stress monitoring, tissue examination and others. This work tries to demonstrate how modern analog front-end integration and embedded design can converge to enable reliable, miniaturized, and energy-efficient biosignal acquisition systems for next-generation wearable technologies.

## 5. Conclusions and Future Work

This work presents a compact and versatile portable platform capable of performing quasi-simultaneous measurements of EDA and BiZ by exploiting the common principles of alternating voltage impedance detection. Although in this study we have focused only on EDA and BiZ measurements, the MsW^2^-v2 platform also integrates state-of-the-art sensors for SPO_2_, temperature, and motion (via a full IMU), all managed by a dual-core microcontroller optimized for low power consumption and wireless data transmission (BLE 5.0). The entire system is implemented on a 1.8 $in^{2}$ PCB and weighs only 25 g including the battery, ensuring portability and comfort for continuous monitoring.

The device has been calibrated using a set of resistive-capacitive laboratory impedances based on the approximate Cole model. Measurement errors detection and correction allows the device to perform measurements with errors below 3 Ω in magnitude and 0.2° in phase over the whole set of laboratory impedances in a range of frequencies from 1 kHz to 200 kHz. Subsequent in vivo experiments confirmed the expected impedance trends with frequency, consistent with the dielectric properties of the first layers of human skin.

The EDA measurement is performed using a constant AC voltage at a very low frequency. This opens up the possibility of performing BiZ measurements at multiple depths within the skin tissue simply by increasing the frequency of the excitation signal, thereby expanding the measurement range with minimal hardware and software complexity. Depending on the intended application of these measurements (activity, mood, tissue characteristics, etc.), the excitation signal frequency must be significantly increased compared to the results shown here, which are merely a basic functional verification. It should be noted that sometimes the impedance value itself is not as important as its variation. In such cases, no further calibration is necessary, and the device described here is ready for use.

## Figures and Tables

**Table 1:** Methodological comparison of research-grade and mass-market wearable devices.

Technical Criterion	Research-Grade Devices				Mass-Market Wearables	
	Sh.3 GSR+	Sh.3 EBio	E4/Em. Plus	MsW^2^-v2	Google Pixel/Fitbit 2	Samsung Galaxy Watch
**Primary Measurement**	EDA (GSR)	BiZ and ECG	EDA (GSR)	EDA + BiZ	cEDA	BiZ
**Configuration**	2 wires	4 wires (tetrapolar)	2 wires	2/4 wires	2 wires	4 wires (indirect)
**Raw Data Access**	Full (μS)	Full	Full (CSV)	Full	Blocked	Blocked
**Sampling Rate**	Adjustable	Adjustable	Fixed (4 Hz)	Adjustable	Very low	On demand
**Electrical Stimulus**	DC	AC	DC	AC	DC	AC

**Table 2:** Summarized Hardware Component Specifications.

IC Model	Measured Variable	Key Technical Specifications
**MAX32666**	Master MCU/BLE	Dual-core microprocessor, integrated BLE 5, microSD storage.
**MAX1555**	Battery management	3.7 V lithium battery charge/discharge controller, low-power.
**MAX32664**	Sensor sub (co-processor)	Optical sensor hub for MAX30101, fast-mode slave $I^{2}C$ interface.
**AD5941**	EDA/BiZ	Low-power AFE, 16-bit ADC, hardware DFT complex impedance, SPI.
**BMI160**	Acceleration/angular velocity	6-axis IMU (16-bit), ±2 to ±16 g, ±125 to $\pm 2000^{\circ }$/s, $I^{2}C$/SPI.
**MAX30101**	SpO_2_/heart rate (HR)	Pulse oximeter module, internal red/IR/freen LEDs, photodetectors.
**MLX90632**	Skin temperature	Non-contact FIR, $50^{\circ }$ FoV, ±0.2 °C medical accuracy, $I^{2}C$ (1.8 V).

**Table 3:** Comparative analysis of integrated circuits for EDA and BiZ measurement.

IC Model	Manufacturer	Frequency Range	EDA/BiZ Uncertainty	Electrodes	Notes/Applications
**AD5941**	AD	0.01 Hz–200 kHz	Error <1% (typ. 1.21%).	Configurable 2- to 4-wire electrodes supported	Flexible AFE. Ideal for research. EDA and multi-frequency BiZ. Low power.
**AFE4500**	TI	5–250 kHz	Error <1% (up to 3kΩ contact impedance).	Supports 2- to 4-wire BiZ measurements	Designed for wearable EDA+BiZ.
**MAX30009**	M/AD	10 Hz–1.3 MHz	Error <±0.5% (between 5 and 50 kHz).	Supports 2- to 4-wire BiZ measurements	Ultra-low power. Very small package. Compact wearables.
**AFE4300**	TI	5–50 kHz	Error ±1% to ±2% (requires external calibration).	Designed for 4-wire BiZ measurements	Used in body composition scales and related applications.

**Table 4:** Set of laboratory impedances and Cole model parameter values.

Impedance	$R_{s}$	$R_{p}$	$C_{p}$
Model	(Ω)	(kΩ)	(nF)
** $Z_{1}$ **	150	220	100
** $Z_{2}$ **	220	560	100
** $Z_{3}$ **	270	1000	22
** $Z_{4}$ **	330	1500	22
** $Z_{5}$ **	470	2200	10
** $Z_{6}$ **	1000	3000	1

**Table 5:** Detailed statistical summary of complex impedance by spectral frequency zones (averaged metrics).

Impedance	Averaged Metric	1–50 kHz	50–100 kHz	100–150 kHz	150–200 kHz
Z1	∥Z∥ Mean Value (Ω)	350.60	151.91	149.61	149.29
	∥Z∥ Mean Error (Ω)	0.4935	0.1611	0.3547	0.4497
	∥Z∥ Type A Uncert. (Ω)	1.0224	0.0186	0.0191	0.0204
	θ Mean Value (°)	−33.03	−6.18	−1.48	1.11
	θ Mean Error (°)	0.0911	0.0436	0.0381	0.0347
	θ Type A Uncert. (°)	0.0152	0.0084	0.0064	0.0080
Z2	∥Z∥ Mean Value (Ω)	459.21	221.58	221.01	220.68
	∥Z∥ Mean Error (Ω)	0.9416	0.0931	0.0811	0.1030
	∥Z∥ Type A Uncert. (Ω)	3.6937	0.0334	0.0290	0.0313
	θ Mean Value (°)	−29.19	−4.74	−1.00	1.20
	θ Mean Error (°)	0.0487	0.0181	0.0197	0.0219
	θ Type A Uncert. (°)	0.0254	0.0059	0.0061	0.0067
Z3	∥Z∥ Mean Value (Ω)	514.42	285.11	274.38	271.28
	∥Z∥ Mean Error (Ω)	2.5890	0.6548	0.5401	0.6134
	∥Z∥ Type A Uncert. (Ω)	0.1002	0.0267	0.0243	0.0261
	θ Mean Value (°)	−48.67	−17.80	−9.77	−5.01
	θ Mean Error (°)	0.1679	0.0954	0.0674	0.0631
	θ Type A Uncert. (°)	0.0062	0.0041	0.0049	0.0053
Z4	∥Z∥ Mean Value (Ω)	1258.91	345.64	333.62	330.75
	∥Z∥ Mean Error (Ω)	0.2882	0.0526	0.0699	0.0763
	∥Z∥ Type A Uncert. (Ω)	0.1147	0.0157	0.0192	0.0326
	θ Mean Value (°)	−48.85	−16.37	−7.92	−3.66
	θ Mean Error (°)	0.0104	0.0085	0.0125	0.0181
	θ Type A Uncert. (°)	0.0034	0.0028	0.0030	0.0046
Z5	∥Z∥ Mean Value (Ω)	2440.80	520.62	487.72	478.93
	∥Z∥ Mean Error (Ω)	0.4905	0.1166	0.1030	0.1560
	∥Z∥ Type A Uncert. (Ω)	0.2921	0.0468	0.0416	0.0475
	θ Mean Value (°)	−56.34	−22.92	−12.29	−6.97
	θ Mean Error (°)	0.0062	0.0076	0.0078	0.0132
	θ Type A Uncert. (°)	0.0024	0.0022	0.0026	0.0041
Z6	∥Z∥ Mean Value (Ω)	8331.18	2448.08	1652.39	1388.25
	∥Z∥ Mean Error (Ω)	1.5988	0.2223	0.2079	0.2583
	∥Z∥ Type A Uncert. (Ω)	1.0590	0.1118	0.0931	0.1037
	θ Mean Value (°)	−79.50	−62.51	−48.14	−37.85
	θ Mean Error (°)	0.0209	0.0038	0.0072	0.0090
	θ Type A Uncert. (°)	0.0054	0.0016	0.0023	0.0034

## Data Availability

The original contributions presented in this study are included in the article. Further inquiries can be directed to the corresponding author.

## Abbreviations

The following abbreviations are used in this manuscript:

AFE	Analog Front End
EDA	Electrodermal Activity
BiZ	Bioimpedance
ST	Skin Temperature
SpO_2_	Blood Oxygen Saturation
IMU	Inertial Measurement Unit
